# Histone Deacetylase Inhibitor Suberoylanilide Hydroxamic Acid Improves Energetic Status and Cardiomyogenic Differentiation of Human Dilated Myocardium-Derived Primary Mesenchymal Cells

**DOI:** 10.3390/ijms21144845

**Published:** 2020-07-08

**Authors:** Rokas Miksiunas, Kestutis Rucinskas, Vilius Janusauskas, Siegfried Labeit, Daiva Bironaite

**Affiliations:** 1 Department of Regenerative medicine, State Research Institute Centre for Innovative Medicine, LT 08406 Vilnius, Lithuania; rokas.miksiunas@gmail.com; 2 Centre of Cardiothoracic Surgery of Vilnius University Hospital Santariskiu Clinic, LT 08406 Vilnius, Lithuania; Kestutis.Rucinskas@santa.lt (K.R.); Vilius.Janusauskas@santa.lt (V.J.); 3 Department of Integrative Pathophysiology, Universitätsmedizin Mannheim, Maybachstr. 14, 68169 Mannheim, Germany; labeit@medma.de; 4 Myomedix Ltd., Im Biengarten 36, 69151 Neckargemuend, Germany

**Keywords:** histone deacetylase inhibitors, dilated cardiomyopathy, cardiomyogenic differentiation, primary mesenchymal cells

## Abstract

Background. In this study the effect of histone deacetylase (HDAC) inhibitor suberoylanilide hydroxamic acid (SAHA) on the energetic status and cardiomyogenic differentiation of human healthy and dilated myocardium-derived mesenchymal stromal cells (hmMSC) have been investigated. Methods. The hmMSC were isolated from the healthy and dilated post-operation heart biopsies by explant outgrowth method. Cell proliferation, HDAC activity, mitochondrial membrane potential, and level of adenosine triphosphate (ATP) were evaluated. The effect of SAHA on mitochondrial parameters has been investigated also by Seahorse XF analyzer and cardiomyogenic differentiation was confirmed by the expression of transcription factor NK2 Homeobox 5 (Nkx2.5), cardiac troponin T and alpha cardiac actin at gene and protein levels. Results. Dilated myocardium-derived hmMSC had almost 1.5 folds higher HDAC activity compared to the healthy cells and significantly lower mitochondrial membrane potential and ATP level. HDAC class I and II inhibitor SAHA improved energetic status of mitochondria in dilated myocardium-isolated hmMSC and increased expression of cardiac specific proteins during 14 days of exposure of cells to SAHA. Conclusions. HDAC inhibitor SAHA can be a promising therapeutic for dilated cardiomyopathy (DCM). Dilated hmMSC exposed to SAHA improved energetic status and, subsequently, cardiomyogenic differentiation. Data suggest that human dilated myocardium-derived MSC still have cardio tissue regenerative potential, which might be stimulated by HDAC inhibitors.

## 1. Introduction

Heart failure is one of the most common heart diseases with over a million hospitalizations annually in the United States and Europe [1]. Dilated cardiomyopathy (DCM), as a leading cause of heart failure, is related to high rates of morbidity and mortality and is the most frequent indication for cardiac transplantation [2]. Patients suffering from dilated cardiomyopathy display reduced thickness of heart wall, increased size of left ventricle chamber, and systolic dysfunction [3]. There are multiple causative factors of DCM, including toxic environmental effects, inflammation, autoimmune disease, viral infections, and others that, in one way or another, affect ventricle blood pumping [4]. Abnormalities in dilated heart are associated with changed physiological parameters, such as ejection fraction, as well as with impaired structural (sarcomere) and metabolic cell functions [5,6]. Treatment of cardiomyopathy is challenging due to the complexity of the disease, different etiology, and a variety of impaired intracellular and extracellular molecular mechanisms [7]. Therefore, to search for new ways to improve functioning of human dilated myocardium is challenging and of particular importance. 

The permanent heart contraction requires a high level of energy. Under the normoxic condition, mitochondria produce more than 95% of adenosine triphosphate (ATP), whereas glycolysis only 5% [8,9]. Though the heart has a relatively low glycogen pool, the rate of glycogen turnover is high and can contribute an extra energy demand for heart contraction [10]. Glucose and fatty acid utilization is also tightly co-regulated and highly relevant to heart failure [11]. It was shown that proper functioning of mitochondria, as an energy supplier, is crucial not only for the stem cell survival but also for regulating their differentiation into diverse cell types, including cardiomyocytes [12,13,14]. The close connection between requirement of energy and proper heart tissue contraction has been also shown at various levels [15]. During various pathological stresses, mitochondria are at high risk and related to cardiac cell death and heart tissue dysfunction, whereas the strengthening of mitochondria to withstand such stresses is directly connected to the better heart tissue regeneration [16]. At the molecular level, failing heart tissue displays decreased mitochondrial respiration, impaired activity of mitochondrial respiratory chain, and oxidative phosphorylation [17,18,19]. It was shown that cardiac energy deficit directly correlates with clinical symptoms of heart failure and the prediction of mortality [20,21]. However, the energetic status of human dilated myocardium-derived hmMSC and the way of its possible improvement are still not clear. 

Histone deacetylases (HDAC) catalyze the removal of acetyl groups from histones and/or targeted proteins, mostly negatively modulate gene expression, and orchestrate the development of various pathological processes within all tissues including the heart [22]. Altogether 18 mammalian HDACs are encoded by distinct genes that, in one way or another, regulate cardiac processes with the most prominent role of HDAC I (HDAC1,2,3,8), IIa (HDAC4,5,7,9,), and IIb (HDAC 6,10) classes [23]. Sirtuins (1–7), III group of histones deacetylases, are structurally and mechanistically distinct from other classes of histone deacetylases (classes I, IIA, IIB, and IV), which have a different protein fold and use zinc (Zn^2+^) as a cofactor. Therefore, sirtuins were not investigated in this study. Firstly, it was shown that HDAC IIa class interacts with myocyte enhancer factor-2, which regulates cardiac hypertrophy [24]. Later on, it was shown that long-term suppression of hypertrophy was associated with reduced morbidity and mortality in patients with hypertension, but not with pathological cardiac remodeling [25]. There is also evidence that HDACs might contribute to the pathological state of heart in rodent models in vivo [26,27]. HDAC inhibitors are increasingly used in chemotherapy and, therefore, their non-specific effects on the heart cells warrant a better understanding as toxicity of chemotherapy often affects the heart most adversely. More recently, low molecular weight HDAC inhibitors have been proposed as not only having anticancer effect but also for changing the epigenetic status of mouse heart tissue, inhibiting inflammatory processes, reducing infarct size in rat ischemia/perfusion mode, and can be proposed as treatment option for diastolic heart failure in animal model studies [27,28]. Before this novel concept can be applied to patients, more in-depth studies are required on human materials, including studies on human cells. 

However, the effect of HDAC inhibitors on the energetic status and cardiomyogenic differentiation of human dilated myocardium-derived MSC has not been evaluated. Therefore, in this study, for the first time, the impact of HDAC I and II class inhibitor suberoylanilide hydroxamic acid (SAHA) on the HDAC activity, level of ATP, mitochondrial membrane potential, and cardiomyogenic differentiation of human healthy and dilated myocardium-derived MSC were investigated. To the best of our knowledge, such effects of HDAC inhibitors on energetic cardioregenerative mechanisms of human dilated myocardium-derived mesenchymal stromal cells so far had not been investigated.

## 2. Results

### 2.1. Isolation and Identification of Healthy and Pathological hmMSCs

**Figure 1:**
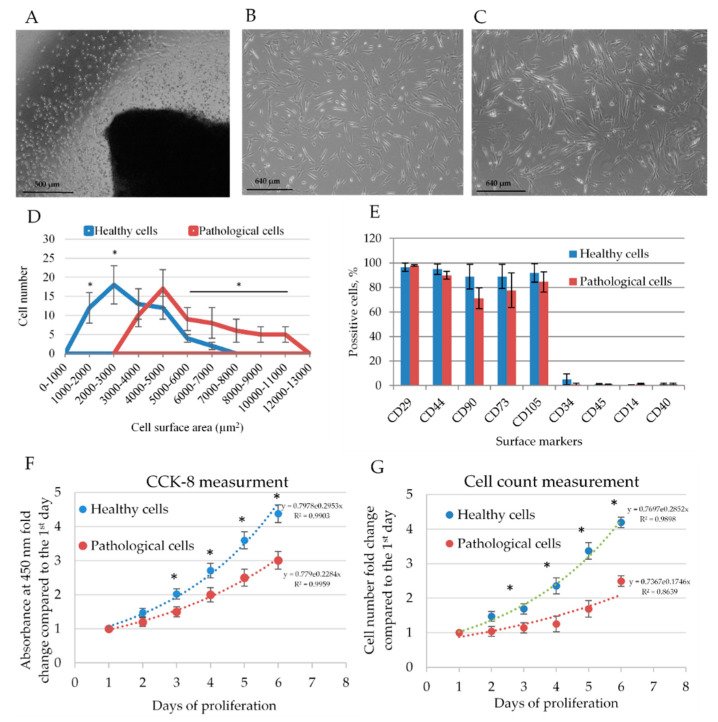
The phenotype and proliferation rate of healthy and pathological human myocardium-derived mesenchymal stromal cells (hmMSC). (**A**) Obtaining of hmMSC from the human heart explants after five days of cultivation, scale bar = 500 μm. Morphology of healthy (**B**) and pathological (**C**) hmMSC, scale bar = 640 μm. (**D**) Graphical representation of attachment area for healthy and pathological cell. The distribution of 60 cells of each type according to the attached area are presented. (**E**) Expression of surface markers on healthy and pathological hmMSC. The proliferation rate of healthy and pathological hmMSC evaluated by metabolic Cell Counting Kit-8 (CCK8) (**F**) and cell-counting method (**G**). Data are shown as mean ± standard deviation (SD). The * *p* ≤ 0.05, *n* = 6 from three experiments calculated by an Excel program. Total adherent surface of different cell types and the minor and major axes of healthy and pathological cells are presented as Supplementary Figure S1.

Human myocardium-derived MSC were successfully isolated by explant outgrowth method from human healthy and dilated myocardium (Figure 1A). Both healthy and pathological hmMSC displayed typical MSC-like morphology. However, the hmMSC isolated from dilated myocardium (Figure 1C) were bigger in size and more flattened compared to the healthy cells (Figure 1B). The calculation of cell attachment surface revealed the biggest part of pathological cells’ population being almost two folds (4000–5000 µm^2^) bigger than the healthy ones (2000–3000 µm^2^) (Figure 1D). In addition, pathological cells were wider on the minor axis. However, cell length on the major axis was quite similar between cell types (Figure S1). 

Both types of isolated hmMSC cells expressed the main MSC surface markers: were positive for Cluster of Differentiation integrin beta-1 (CD29), homing cell adhesion molecule (CD44), thymocyte differentiation antigen 1 (CD90), ecto-5′-nucleotidase (CD73), and endoglin (CD105) and negative for protein tyrosine phosphatase, receptor type, C (CD45), macrophage protein, which binds lipopolysaccharide (CD14), costimulatory protein found on antigen-presenting cells (CD40) (Figure 1E) and in early passages expressed low amounts of cell-cell adhesion factor (CD34). The dilated myocardium-derived MSC had slightly lower levels of measured cell surface markers. 

The proliferation of healthy and pathological hmMSC was measured using Cell Counting Kit-8 (CCK8) and cell-counting methods (Figure 1E). Healthy hmMSC proliferated almost two folds faster than pathological hmMSC (Figure 1E). The difference in proliferation rate between healthy and pathological hmMSC was similar measured by both methods. It revealed that the metabolic way of cell counting by CCK-8 corresponded to cell number. 

### 2.2. Energetic Profile of Healthy and Pathological hmMSCs

**Figure 2:**
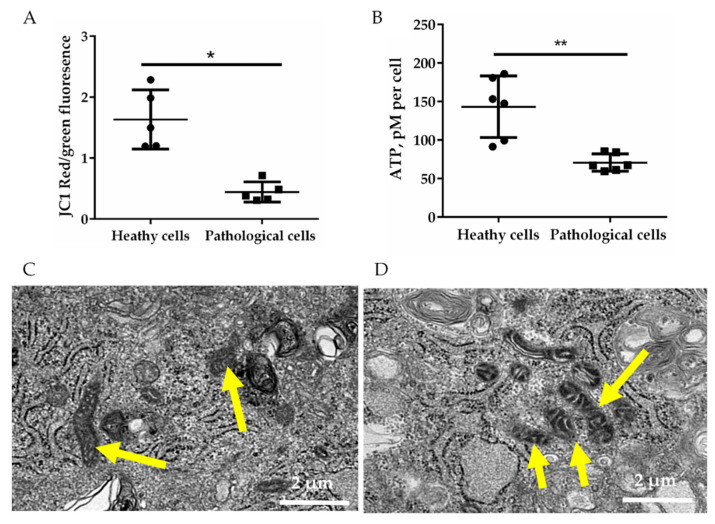
Energetic status of healthy and pathological hmMSC. (**A**) Mitochondrial membrane potential measured by 5,5′,6,6′-Tetrachloro-1,1′,3,3′-tetraethyl-imidacarbocyanine iodide (JC1) dye. (**B**) Level of ATP in healthy and pathological hmMSCs (picomoles (pM) of adenosine triphosphate (ATP) per cell). Representative micrographs of electron microscope of healthy (**C**) and pathological (**D**) hmMSC are shown, scale bar = 2 µm. Yellow arrows indicate mitochondria. Data are shown as mean ± standard deviation (SD). The * *p* ≤ 0.05, ** *p* ≤ 0.01, *n* = 5 from three experiments. Student t test was calculated by Graphpad Prism 6 program.

Further, in order to evaluate mitochondrial membrane potential, the green and red fluorescence of 5,5′,6,6′-Tetrachloro-1,1′,3,3′-tetraethyl-imidacarbocyanine iodide (JC1) within healthy and pathological hmMSC was measured by flow cytometry (Figure 2A). Cells with active mitochondrial membrane potential accumulate a higher level of JC1, resulting in red fluorescence of JC1 aggregates, whereas mitochondria with lower membrane potential have green fluorescence of monomeric JC1. Data show that healthy hmMSC had three folds’ more of active mitochondria compared to the pathological cells (Figure 2A). The lower level of active mitochondria in pathological hmMSC showed accordingly lower ATP production. The total level of ATP was approximately two-fold lower in pathological cells compared to the healthy ones (Figure 2B). The morphology of pathological cell mitochondria, analyzed by the electron microscope, was slightly bigger and/or swollen (Figure 2D) compared to the healthy hmMSC (Figure 2C). Additionally, dilated myocardium-derived cells had larger and more prominent vacuoles typical for the dilated myocardium than healthy cells.

**Figure 3:**
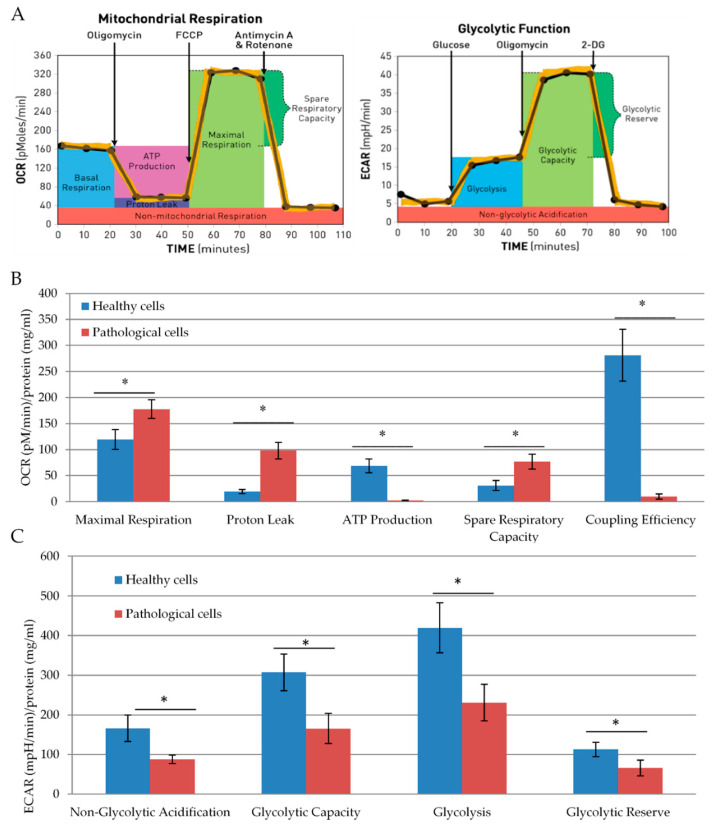
Mitochondrial respiration and glycolytic capacity of healthy and pathological primary hmMSC. (**A**) Schematic representation of oxygen consumption and glycolytic acidification assays. (**B**) Mitochondrial activity of healthy and pathological hmMSC. (**C**) Glycolytic capacity of healthy and pathological hmMSC. Oxygen consumption (pM) and glycolytic acidification (milli pH (mpH)/min) values were normalized to protein level (mg/mL). Data are shown as mean ± standard deviation (SD). The * *p* ≤ 0.05, *n* = 3 from two experiments. Student t test was calculated by an Excel program.

Furthermore, we performed a more detailed analysis of mitochondrial activity of healthy and pathological hmMSC by Seahorse XF analyzer. Seahorse evaluates mitochondria function and glycolysis by measuring the oxygen consumption rate and extracellular acidification, respectively (Figure 3A). Seahorse data confirmed previous observations that pathological cells had a two-fold lower amount of ATP than healthy cells. Maximal respiration was significantly higher in pathological cells as compared to the healthy cells (177.6 ± 17 and 120 ± 18). However, due to the higher proton leak (98.1 ± 5.1 and 19.23 ± 4.0), their respiration was much worse coupled (2.7 ± 0.6 and 68.9 ± 13.0) with oxidative phosphorylation than in healthy hmMSC (Figure 3B). Despite that, the pathological cells had a higher level of spare respiratory activity compared to the healthy cells (76.7 ± 14.5 and 30 ± 10.5), which could be properly used.

Healthy hmMSC were more glycolytically active compared to the pathological hmMSC (Figure 3C). Glycolytic capacity and glycolysis were almost twice higher in healthy cells compared to the pathological cells. High glycolytic rate is usually related to the more rapid cell proliferation, so this might explain the higher proliferative capacity of healthy hmMSC compared to the pathological ones. 

### 2.3. Histone Deacetylase Activity and Effect of SAHA on Healthy and Pathological hmMSC

**Figure 4:**
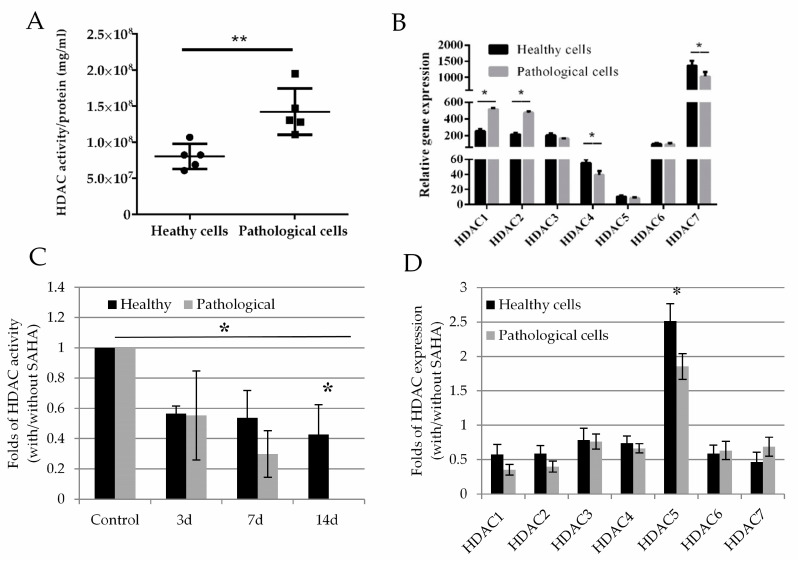
HDAC activity and gene expression profile in healthy and pathological hmMSC. (**A**) HDAC activity in healthy and pathological hmMSC (arbitrary fluorescence units/per protein (mg/mL), *n* = 5. (**B**) Relative expression (2^−ΔCT^) of *HDAC1–7* genes in healthy and pathological hmMSC, *n* = 3. Changed HDAC activity (**C**) and *HDAC1–7* gene expression (2^−ΔΔCT^) (**D**) in healthy and pathological cells after exposure to 1 µM of SAHA for three days, *n* = 3. Gene expression was analyzed as follows: expression = 2^−ΔCt^ × 100,000, where ΔCt = (Ct_target gene_ − Ct_reference gene_); relative expression = 2^−ΔΔCt^, where ΔΔCt is a subtraction of ΔCt of control cells from ΔCt of cells treated with SAHA. Data are shown as mean ± standard deviation (SD). The * *p* ≤ 0.05, ** *p* ≤ 0.01, *n* = 3–5 from 3 experiments. Student *t*-test was calculated by an Excel and Graphpad Prism 6 programs.

To evaluate HDAC activity, we used the fluorescent HDAC substrate (S)-tert-Butyl (6-acetamido-1-((4-methyl-2-oxo-2H-chromen-7-yl)amino)-1-oxohexan-2-yl)carbamate (BOC-Ac-Lys-AMC). Results revealed that pathological cells had 1.5-fold higher total HDAC activity compared to the healthy hmMSC (Figure 4A). At the gene level, pathological cells also displayed two-fold higher expression of *HDAC1* and *HDAC2* compared to the healthy cells. Meanwhile, the levels of *HDAC4* and *HDAC7* were lower in pathological cells. The levels of *HDAC3*, *HDAC5*, and *HDAC6* were relatively similar between healthy and pathological hmMSC (Figure 4B). Moreover, the expression level of *HDAC5* gene was lowest in healthy (10.5 ± 1.2) and pathological (8.2 ± 0.8) cells, while both types of the cells showed the highest expression level of *HDAC7* (healthy cells 1362.8 ± 123.8, pathological cells 1024.2 ± 112.2). 

Both healthy and pathological cell types, exposed to 1 µM of SAHA for 14 days, reduced levels of HDAC activity (Figure 4C). Pathological cells were more sensitive to 1 µM of SAHA, i.e., HDAC activity was not detectable on the 14th day after exposure of pathological hmMSC to SAHA (Figure 4C). The expression levels of *HDAC1, HDAC2, HDAC3, HDAC4, HDAC6*, and *HDAC7* genes in healthy and pathological cells after exposure to SAHA for three days were significantly suppressed (Figure 4D shows folds of *HDACs* expression with and without SAHA)). The expression level of *HDAC5* in both types of the cells at the control level was very low compared to the other *HDACs* (Figure 4B), but showed some upregulation after expression to SAHA (Figure 4D). It can be related to the compensatory mechanism of activity between various types of HDACs. This part needs more detail investigations.

### 2.4. The Impact of SAHA on Bioenergetics of Healthy and Pathological Cells

**Figure 5:**
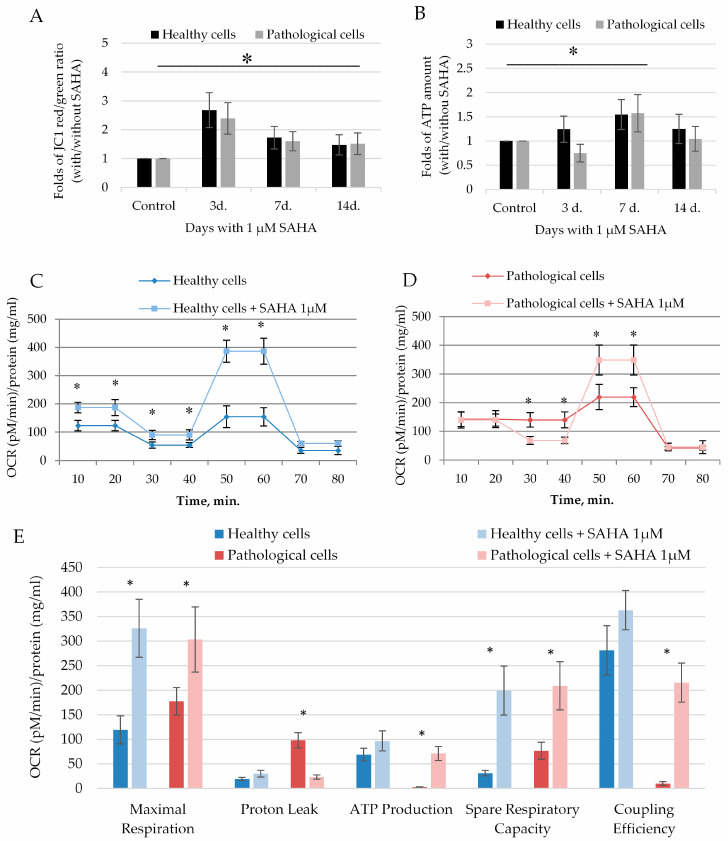
SAHA enhances the energetic status of healthy and pathological hmMSC. Mitochondrial membrane potential (**A**) and ATP production (**B**) in healthy and pathological hmMSCs exposed to 1 µM of SAHA for three days. Changes of oxygen consumption rate (OCR) ((pM)/protein (mg/mL)) in healthy (**C**) and pathological (**D**) cells and calculated mitochondrial parameters (**E**) after exposure to SAHA. Data are displayed as fold of changes with and without SAHA. Control–cells of both types not affected by SAHA. Data are shown as mean ± standard deviation (SD). The * *p* ≤ 0.05, *n* = 3 from 3 experiments. Student *t*-test was calculated by an Excel program.

Further, we investigated whether SAHA can affect the energetic status of healthy and pathological hmMSC. SAHA significantly activated mitochondrial membrane potential in both healthy and pathological hmMSC (Figure 5A). The most significant impact on mitochondrial membrane potential was observed at the third day of exposure to SAHA: For healthy (2.6 ± 0.6 fold) and pathological cells (2.3 ± 0.5 fold) compared to not affected cells (Figure 5A). The impact of SAHA on ATP production was observed later: ATP production in healthy and pathological cells increased by 1.57 ± 0.3 and 1.54 ± 0.3 fold at the seventh day of exposure to SAHA (Figure 5B). Data show that cells, first of all, need to improve functioning of mitochondria and then start to increase ATP production, which is needed for the cardiomyogenic differentiation. 

The more detailed investigation of SAHA effect on mitochondrial capacity parameters by Seahorse analyzer confirmed that both healthy and pathological hmMSC significantly increased oxygen consumption after incubation with 1 µM of SAHA during three days (Figure 5C,D). The most promising effect of SAHA was on pathological hmMSC (Figure 5E). SAHA significantly reduced pathological cells’ proton leak from 98.1 ± 15.5 to 23.2 ± 4.2 and increased ATP production from 2.7 ± 0.4 to 71.1 ± 20.4, activated maximal respiration from 177.6 ± 27.6 to 303.4 ± 66.2, and improved spare respiratory capacity from 76.7 ± 17.1 to 209.1 ± 49.1 and coupling efficiency from 9.9 ± 4.1 to 215.5 ± 40.5 (Figure 5E). The similar, but less prominent, impact of SAHA was observed on healthy cells: It significantly improved maximal respiration from 119.3 ± 28.5 to 326.1 ± 58.9 and spare respiratory capacity from 31.2 ± 5.5 to 199.2 ± 49.1 (Figure 5E). Other parameters, such as ATP production and coupling efficiency, were slightly improved in healthy cells. 

Data show that SAHA improved mitochondrial membrane functioning and efficient oxygen consumption that resulted in increased production of ATP in both types of the cells, with particular positive effect on pathological cells. 

**Figure 6:**
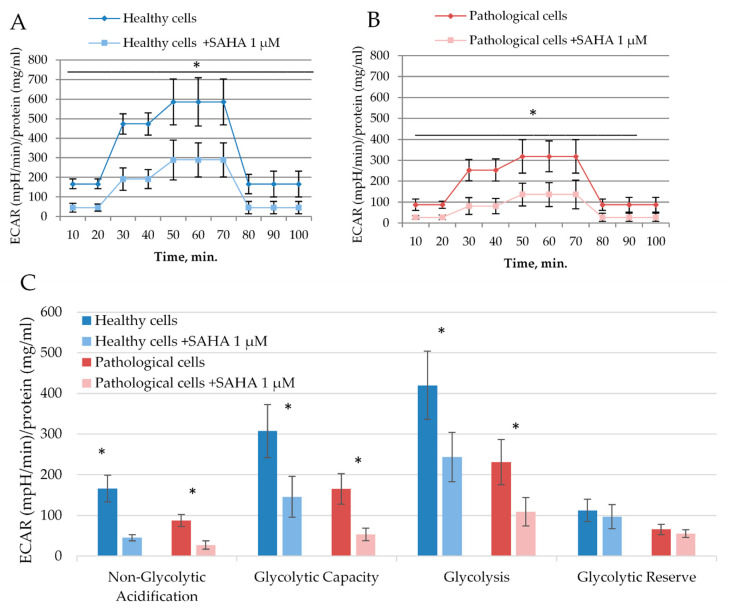
The effect of SAHA on glycolysis of healthy and pathological hmMSC. Decrease of extracellular acidification rate (ECAR) ((mpH/min)/protein (mg/m)) in healthy cells (**A**) and pathological (**B**) cells after exposure to 1 µM of SAHA for three days. (**C**) Quantification of glycolytic stress parameters in healthy and pathological hmMSC with and without SAHA. Data are shown as mean ± standard deviation (SD). The * *p* ≤ 0.05, *n* = 3 from three experiments. Student *t*-test was calculated by an Excel program.

The effect of SAHA was also investigated on glycolysis of healthy and pathological hmMSC (Figure 6A,B). Data show that healthy and pathological cells exposed to 1 µM of SAHA for three days resulted in reduced glycolysis (Figure 6C). SAHA reduced glycolytic stress parameters in healthy cells: Non-glycolytic acidification from 166.1 ± 32.7 to 45.4 ± 7.7, glycolytic capacity from 307.1 ± 65.3 to 146.1 ± 50.3, and glycolysis from 419.7 ± 83.9 to 243.3 ± 60.1. A similar effect of SAHA was on the pathological cells: Non-glycolytic acidification decreased from 87.5 ± 14.4 to 27.4 ± 10.6, glycolytic capacity from 165.3 ± 37.1 to 53.5 ± 15.2, and glycolysis from 231.1 ± 55.3 to 109.1 ± 35.1.

### 2.5. The Impact of SAHA on Cardiomyogenic Differentiation

**Figure 7:**
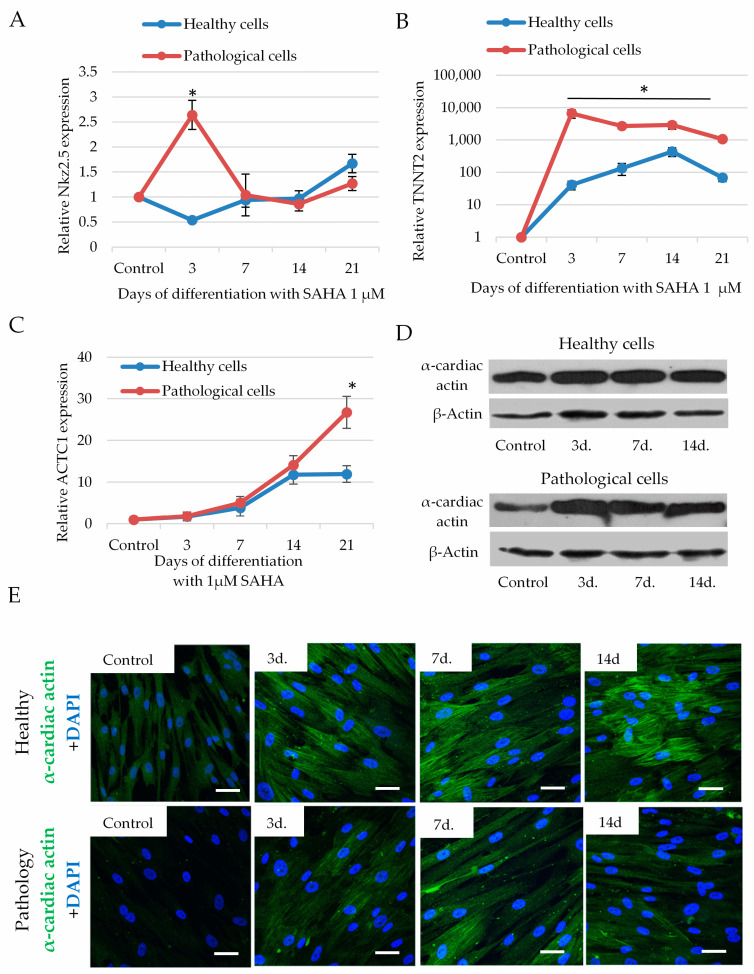
The impact of SAHA on the levels of transcription factor transcription factor NK2 Homeobox 5 (*Nkx2.5*), cardiac troponin T (*TNNT2*) and alpha cardiac actin (*ACTC1*), in healthy and pathological hmMSC. (**A**) Expression of *Nkx2.5* gene (2^−ΔΔCT^) in healthy and pathological cells during 3–21 days of cultivation with 1 µM of SAHA. (**B**) Expression of *TNNT2* gene (2^−ΔΔCT^) in healthy and pathological cells during 3–21 days of cultivation with 1 µM of SAHA. (**C**) Expression of *ACTC1* gene (2^−ΔΔCT^) in healthy and pathological cells during 3–21 days of cultivation with 1 µM of SAHA. (**D**) Level of alpha cardiac actin protein compared to beta actin in healthy and pathological cells after exposure to 1 µM of SAHA analyzed by Western blotting. (**E**) Immunocytochemical micrographs of alpha cardiac actin levels in healthy and pathological cells after exposure to 1 µM of SAHA for 3–14 days, scale bar = 20 μm. Control-healthy and pathological cells without SAHA. Gene expression was analyzed as follows: expression = 2^−ΔCt^ × 100,000, where ΔCt = (Ct_target gene_ − Ct_reference gene_); relative expression = 2^−ΔΔCt^, where ΔΔCt is a subtraction of ΔCt of control cells from ΔCt of cells treated with SAHA. Data are shown as mean ± standard deviation (SD). The * *p* ≤ 0.05, *n* = 3 from three experiments. Student *t*-test was calculated by an Excel program. (**D**) and (**E**)—representative blots and micrographs are shown.

To investigate efficiency of cardiomyogenic differentiation, we measured expression of transcription factor transcription factor NK2 Homeobox 5 (*Nkx2.5*) and structural proteins: Cardiac troponin T (*TNNT2*) and alpha cardiac actin (*ACTC1*) after exposure of cells to 1 µM of SAHA for 21 days. SAHA significantly increased expression of *Nkx2.5* in pathological cells, which was highest on the third day (2.64 ± 0.24 fold compared to not affected cells), which, later on, decreased (Figure 7A). The expression of *TNNT2* in pathological cells was also highest at the third day after of exposure to SAHA (6700 ± 2010 fold compared to not affected cells) and slightly decreased during the next 14–21 days (Figure 7B). In the healthy cells, the highest expression of *Nkx2.5* and *TNNT2* was observed later on, at 14–21 days of exposure to SAHA. Data suggest that activation of transcription factor *Nkx2.5* and *TNNT2* have a direct connection and pathological cells more quickly responded to the SAHA stimulus than the healthy cells. 

Additionally, SAHA promoted expression of *ACTC1* in both healthy and pathological hmMSC that was slower compared to the *TNNT2*: The highest upregulation of *ACTC1* gene for the pathological cells was at 21 days (26.7 ± 4.3 folds compared to the control cells), whereas for the healthy cells the expression of *ACTC1* became stable at 14–21 days (14.1 ± 2.6 folds compared to the control cells) (Figure 7C). At the protein level, SAHA also showed upregulation of alpha cardiac actin in both pathological and healthy cells analyzed by Western blotting (Figure 7D) and immunocytochemistry (Figure 7E). The slight expression of alpha cardio actin at control level can be related to the cardio tissue specificity of adult heart-derived cells, which was higher in the healthy cells compared to the pathological (Figure 7E). Raw data of *Nkx2.5*, cardiac troponin T (*TNNT2*), and alpha cardiac actin (*ACTC1)* genes’ expression at control level is shown in Figure S2.

## 3. Discussion

Dilated cardiomyopathy remains to be responsible for 40–50% of cases of heart failure in humans with poor outcomes [29]. The molecular mechanisms involved in the development of dilated cardiomyopathy, as well as toxic DCM causes, are complex and multifactorial including changed myocardial cell bioenergetics, calcium handling, gene expression profile, impaired contractility of cardiomyocytes, and others [30,31,32,33]. Therefore, the investigation of DCM causing molecular mechanisms and relevant prevention means is of particular importance. It was already agreed that heart tissue is not a post-mitotic organ, constituting cardiomyocytes, fibroblasts (or mesenchymal stromal cells, MSC), and endothelial cells, and having some, albeit limited, regenerative capacity [34,35]. Since the adult human heart has a very limited number of human receptor tyrosine kinase (c-kit) positive stem cells [36], other types of cells residing in the heart such as human myocardium-derived MSC react to the external stimuli and can be successfully used for cardio regenerative purposes [36,37]. In this study, the HDAC activity and energetic potential of mesenchymal stromal cells (MSC), also named as medicinal signaling cells [38], derived from human healthy and dilated myocardium biopsies and their response to HDAC class I/II inhibitor SAHA were investigated.

Activities of histone deacetylases and their impact on the heart tissue functioning were investigated in multiple rodents’ heart tissue disease models, i.e., it was shown that overexpression of HDACs’ class I members HDAC1 and HDAC2 can contribute to the pathological heart state by promoting fibrosis, inflammation, and, later on, heart failure in rat post-myocardial infarction (MI) and rat cardiac hypertrophy models in vivo [26,39], and suppression of HDAC I group activity was shown to antagonize mice cardiac hypertrophy by reducing expression of stress proteins and interstitial fibrosis [40]. HDACs’ class II sometimes can have controversial roles: The overexpression of *HDAC4* in mouse ischemia/reperfusion model resulted in reduced myocardium recovery [41] and excessive activity of HDAC6 in diabetic rats was also related to increased vulnerability to ischemia/reperfusion injury [42]. However, HDAC5 was shown to play an integral role in cardiac development, suppression of oxidative stress, and heart hypertrophy [43,44,45], while HDAC7 maintained vascular integrity during early mouse heart development and participated in endothelial cell function [46]. 

At the cellular level, HDAC class I members HDAC 1 and HDAC2 are exclusively nuclear and HDAC3 is nearly always localized in the nucleus [47]. Therefore, the significant increase of HDAC1 and HDAC2 pathological hmMSC could have a stronger impact on DCM, whereas their response to SAHA could more significantly increase expression of cardiomyogenic genes than class II HDACs. Since class II HDACs shuttle in and out of the nucleus in response to cellular signals, they can be more responsible for the cardiac physiology genes than for the cell functioning [48]. Moreover, inhibition of HDAC showed enhanced oxidative metabolism in skeletal muscle and adipose tissue of diabetic mice [49]. The overexpression of *HDAC1* and *HDAC2* genes in dilated myocardium-derived hmMSC, compared to the healthy hmMSC, was significantly higher, whereas the expression levels of class II HDACs were relatively similar between healthy and pathological hmMSC. However, even if there is no concrete evidence of involvement of HDAC7 in heart failure, the lower level in pathological hmMSC, compared to the healthy cells, suggests vascular abnormalities seen in dilated cardiomyopathy. Data suggest that HDACs’ class I members HDAC 1 and HDAC2 can more contribute to the phenotype of dilated myocardium-derived hmMSC and their regenerative potential after exposure to HDAC inhibitors, compared to the class II HDACs. There is also a possibility that overexpression of some HDAC II members in cardiac cells is related to worse cardioregeneration potential. More detailed studies in this field are needed.

Most of the studies investigating HDAC inhibitor SAHA have been done with various cancer cell lines arresting cancer cell growth mainly by blocking their glycolysis pathways [50,51,52]. In terms of heart functioning, studies with SAHA have been done mostly on rodent heart disease models, rabbit ischemia/reperfusion injury, and rat hypertensive cardiomyopathy [27,53]. At cellular level, SAHA promoted expression of cardiomyogenic specific proteins in rat bone marrow-derived MSC and human dental follicle cells [54,55]. Since, close connection between impairment of mitochondrial respiratory chain and reduced ATP generation has been confirmed in late stage of DCM patients [56,57], it was interesting to investigate the impact of SAHA not only on HDAC activity but also on the energetic status of human healthy and dilated myocardium-derived hmMSC, i.e., whether or not SAHA can suppress HDAC activity and improve energetic status of hmMSC, particularly derived from the dilated myocardium. Dilated myocardium-derived hmMSC, compared to the healthy cells, had almost twice lower mitochondrial membrane potential and ATP level that was significantly improved by HDAC inhibitor SAHA: Dilated myocardium-derived cells, after exposure to SAHA for three days, increased oxygen consumption and ATP production and significantly reduced proton leak due to the better coupling of respiration and oxidative phosphorylation. Improved mitochondrial functioning and cell energetic status support better expression of cardiomyogenic differentiation specific transcription factors (*Nkx2.5*) and structural proteins (*TNNT2* and *ACTC1*) at gene level. Additionally, alpha cardiac actin expression was increased at protein level as well. The full maturation (as cell contraction function) of differentiated adult human myocardium-derived MSC after exposure to SAHA has not been detected. However, the full maturation of cardiomyocyte is a very complex process and requires expression of specific genes, electrophysiology and Ca^2+^ handling, metabolic changes, synchronous contraction, and other [58]. Although current technologies allowed for efficient cardiomyogenic differentiation of human pluripotent cells in vitro, these cardiomyocytes still exhibit immature phenotypes [59,60]. Despite that, the increased expression of cardiomyogenic differentiation specific genes and proteins in adult human heart-derived MSC, particularly in dilated myocardium, after the exposure to SAHA is a first step in demonstrating cardio differentiation processes in vitro. 

Based on multiple heart failure models, due to different disease etiologies, there is no conclusive correlation between glycolysis and contractile function in heart failure [9]. However, it was shown a higher glucose utilization rate in patients suffering from idiopathic dilated cardiomyopathy [61]. Measuring of glycolysis in vivo displays glycolytic condition of whole tissue rather than in individual cell. Although some successful attempts to measure glycolysis in rat cardiomyocytes were done [62], there are no data of such measurements in human healthy and especially in dilated myocardium-derived mesenchymal cells. Our results show that healthy cells had a slightly higher glycolytic rate compared to the pathological cells that could explain their better proliferation. SAHA significantly downregulated glucose metabolism and glycolytic processes in both healthy and pathological cells but increased mitochondrial capacity and the typical oxidative phosphorylation process necessary for the successful differentiation process. Based on these findings, we can suggest that switching of energetic balance from glycolytic toward mitochondrial oxidation improves cardiomyogenic differentiation of both healthy and dilated myocardium-derived mesenchymal cells with the prominent effect on dilated myocardium cells. 

## 4. Materials and Methods

### 4.1. Isolation and Growth of hmMSC

Cells were isolated from the post-operation heart muscle biopsy following previously described protocols with some modification [63]. Briefly, the human ventricle myocardium specimens’ heart biopsy specimens were immediately stored on ice and transported to the laboratory, were cut into 1–3-mm^3^ fragments, washed with PBS (Sigma Aldrich, St. Louis, MO, USA) with 2% of antibiotics (Thermo Fisher Scientific, Waltham, MA, USA), and partially digested with 0.25% trypsin-EDTA solution (Thermo Fisher Scientific, Waltham, MA, USA). Tissue fragments were cultured as tissue explants on fibronectin (PeproTech, Rocky Hill, NJ, USA) (2 µg/mL, coated six-well plates in IMDM media (Thermo Fisher Scientific, Waltham, MA, USA) supplemented with 20% FBS (Thermo Fisher Scientific, Waltham, MA, USA) and 100 U/mL penicillin G, 100 U/mL streptomycin (Thermo Fisher Scientific, Waltham, MA, USA) at 37 °C and 5% CO_2_. Approximately in 1–4 weeks, a layer of spindle-like, loosely attached cells emerged that were lifted with diluted trypsin and transferred to a 75-cm^2^ flask coated with gelatin (AppliChem GmbH, Darmstadt, DEU) for further growth. Remaining explants were transferred to the plates coated with the fibronectin for the further formation of cardiac outgrowth. DCM was identified by cardiologists according to the weak ventricle functioning (ejection fraction < 20%) and diastolic diameter higher than 5.5 cm and other clinical parameters. Cells of up to 4–5 passage were used.

### 4.2. Flow Cytometry

The cell monolayer was trypsinized for 5 min at 37 °C. For nonspecific interactions, cells were blocked with 1% BSA (Sigma Aldrich, St. Louis, MO, USA) for 30 min on ice. After blocking, cells were washed and incubated with specific antibodies and respective isotype controls for 30 min on ice: Cluster of Differentiation integrin beta-1 (CD29-ImmunoglobulinG1-Allophycocyanin (1A-219-T100, Exbio, Praha, Czech Republic)), homing cell adhesion molecule (CD44-ImmunoglobulinG2b-Fluorescein isothiocyanate (555478, BD Biosciences, San Jose, CA, USA)), thymocyte differentiation antigen 1 (CD90-ImmunoglobulinG1-Fluorescein isothiocyanate (328108, BioLegend, San Diego, CA, USA)), ecto-5′-nucleotidase (CD73-ImmunoglobulinG1-Fluorescein isothiocyanate (561254, BD Biosciences, San Jose, CA, USA)), endoglin (CD105-ImmunoglobulinG1-Allophycocyanin (MHCD10505, Thermo Fisher Scientific, Waltham, MA, USA)), cell-cell adhesion factor (CD34-ImmunoglobulinG1-Fluorescein isothiocyanate (1F-297-T100, Exbio, Praha, Czech Republic)), protein tyrosine phosphatase, receptor type, C (CD45-ImmunoglobulinG2a-Fluorescein isothiocyanate (sc-70686, Santa Cruz Biotechnology, Dallas, TX, USA)), macrophage protein, which binds lipopolysaccharide (CD14-ImmunoglobulinG2a-Allophycocyanin (301808, BioLegend, San Diego, CA, USA)), and costimulatory protein found on antigen-presenting cells (CD40-ImmunoglobulinG1-Allophycocyanin (555591, BD Biosciences, San Jose, CA, USA)). After staining with antibodies, cells were washed twice with PBS and 1% BSA. Flow cytometry was performed with BD FACSAria II flow cytometer (BD Biosciences, San Jose, CA, USA). 

### 4.3. Measurement of Cell Proliferation

Cells (5 × 10^3^/well) were seeded in 24-well plates. The proliferation of healthy and pathological cells was continuously measured for six days by CCK-8 kit as suggested by manufacturers (Dojindo, Kumamoto, Japan). Briefly, 10 µL of CCK8 was added to 300 µL of changed growth media, the plate was incubated for 3 h at 37 °C, and absorption was measured at 450 nm using SpectraMax i3 (Molecular Devices, San Jose, CA, USA) spectrophotometer. In parallel, cells were stained with trypan blue (Sigma Aldrich, St. Louis, MO, USA) and counted by cell fast read counter (Biosigma, Cona, ITA).

### 4.4. HDAC Activity Assay

Cells were extracted with HDAC reaction buffer (50 mM Tris HCl pH = 7.5, 5% glycerol, 0.3% Triton-100, 50 mM NaCl) and frozen at −80 °C for later use. Thawed samples were centrifuged at 20,000× *g* for 15 min at +4 °C and protein concentration was determined with Pierce™ Modified Lowry Protein Assay Kit (Thermo Fisher Scientific, Waltham, MA, USA). For each HDAC activity reaction, 25–50 µg of protein lysate was mixed with 30 µM of HDAC activity substrate (S)-tert-Butyl (6-acetamido-1-((4-methyl-2-oxo-2H-chromen-7-yl)amino)-1-oxohexan-2-yl)carbamate (Boc-Lys(Ac)-AMC) (Sigma Aldrich, St. Louis, MO, USA) in the reaction buffer (50 mM Tris HCl pH = 8, 100 mM NaCl) and incubated for 30 min at 30 °C. The reaction was stopped with 10 mg/mL trypsin and 25 µM SAHA (Sigma Aldrich, St. Louis, MO USA). Fluorescence was measured at 325/395 nm using spectrophotometer SpectraMax i3 (Molecular Devices, San Jose, CA, USA).

### 4.5. ATP Activity Assay

Cells (3 × 10^3^/well) were seeded in a white, clear-bottom, 96-well plate. ATP measurements were done by ATPlite 1step Luminescence Assay, adding 100 µL of ATPlite 1step solution to each well as recommended by manufacturers (PerkinElmer, Waltham, MA, USA). Luminescence was measured immediately with SpectraMax i3 (Molecular Devices, San Jose, CA, USA) spectrophotometer. ATPlite 1step Luminescence Assay System buffer immediately lysed cells to evaluate the total ATP amount. Before the measurement, samples were centrifuged at 20,000× *g* for 15 min at +4 °C. For ATP measurements in healthy and pathological hmMSC, luminescence was normalized to the cell number (pM of ATP per cell) using ATP standard curve. ATP level in the cells incubated for 3, 7, and 14 days with SAHA was normalized to protein level by lysing cells with lysis buffer (50 mM Tris HCl pH = 6.8, 10% glycerol, 1% SDS) on a separate 96-well plate and measuring with Pierce™ Modified Lowry Protein Assay Kit, (Thermo Fisher Scientific, Waltham, MA, USA). 

### 4.6. Measurement of Mitochondrial Membrane Potential

Cells (10^3^/well) were seeded in a white, clear-bottom, 96-well plate. Cells were incubated with 2 µM of JC1 (Thermo Fisher Scientific, Waltham, MA, USA) for 30 min at 37 °C. Red and green fluorescence was measured with SpectraMax i3 (Molecular Devices, San Jose, CA, USA) spectrophotometer by scanning each of the 96 wells. Mitochondrial potential was evaluated, dividing red and green fluorescence. The mitochondrial potential was also evaluated with flow cytometry without SAHA (Sigma Aldrich, St. Louis, MO, USA). Briefly, trypsinized cells were washed with PBS and incubated with 2 µM of JC1 (Thermo Fisher Scientific, Waltham, MA, USA) for 30 min at 37 °C. After staining, cells were washed twice with PBS and 1% BSA. Flow cytometry was performed with BD FACSAria II flow cytometer (BD Biosciences, San Jose, CA, USA). 

### 4.7. Real Time PCR (RT-PCR)

RNA was purified approximately from 2 × 10^5^ cells using GeneJet^TM^ RNA purification kit (Thermo Fisher Scientific, Waltham, MA, USA). A total of 500 ng of RNA was incubated with DnaseI (Thermo Fisher Scientific, Waltham, MA, USA) for 30 min at 37 °C, and then the reaction was stopped by adding 50 mM of EDTA and heating for 5 min at 65 °C. Reverse transcription was performed with High Capacity CDNA reverse transcription kit according to the manufacturer’s recommendations (Thermo Fisher Scientific, Waltham, MA, USA). Real-time PCR was performed in triplicate using the 2× Maxima Probe qPCR Master Mix (Thermo Fisher Scientific, Waltham, MA, USA) on an AriaMx Real-Time PCR Machine (Agilent Technologies, Santa Clara, CA, USA) with an annealing temperature of 60 °C. Following that, Taqman primers (Thermo Fisher Scientific, Waltham, MA, USA) were used to measure target gene expression: *HDAC1* (Hs02621185_s1), *HDAC2* (Hs00231032_m1), *HDAC3* (Hs00187320_m1), *HDAC4* (Hs01041648_m1), *HDAC5* (Hs00608351_m1), *HDAC6* (Hs00997427_m1), *HDAC7* (Hs01045870_m1), *ACTC1* (Hs01109515_m1), *TNNT2* (Hs00943911_m1), *NKX2.5* (Hs00231763_m1), beta (β)-actin *(ACTB)* (Hs01060665_g1), and glyceraldehyde-3-phosphate dehydrogenase (*GAPDH)* (Hs02786624_g1). The expression of each gene was normalized to the average of *ACTB* and *GAPDH* reference genes’ expression. Gene expression signals were analyzed using AriaMx 1.71 software (Agilent Technologies, Santa Clara, CA, USA) as follows: eexpression = 2^−ΔCt^ × 100,000, where ΔCt = (Ct_target gene_ − Ct_reference gene_); relative expression = 2^−ΔΔCt^, where ΔΔCt is a subtraction of ΔCt of control cells from ΔCt of cells treated with SAHA, Ct is the threshold cycle. Gene expression was displayed as 2^−ΔCT^ × 10,000 in control cells and 2^−ΔΔCT^ after exposure cells to SAHA.

### 4.8. Measurement of Metabolic Activity with Seahorse

We used Seahorse XFp Analyzer (Agilent Technologies, Santa Clara, CA, USA) to measure oxygen consumption rate (OCR) and extracellular acidification rate (ECAR) in healthy and pathological cells. Cells were seeded in Seahorse XFp culture plates at 20,000 cells per well one day before measurement. Seahorse medium (Agilent Technologies, Santa Clara, CA, USA) was prepared according to the manufacturer’s recommendations. For OCR measurements, the final concentrations of 10 mM glucose, 2 mM glutamax, and 1 mM sodium pyruvate were used (Agilent Technologies, Santa Clara, CA, USA). Mitochondrial parameters were measured by sequential addition of 10 µM oligomycin, 20 µM carbonyl cyanide 4-(trifluoromethoxy)phenylhydrazone (FCCP), and 5 µM rotenone (Agilent Technologies, Santa Clara, CA, USA). For ECAR measurements, 1 mM glutamine was added to the Seahorse medium. Glycolytic stress was evaluated by sequential addition of 100 mM glucose, 50 µM oligomycin, and 500 mM 2-deoxyglucose (DG) (Agilent Technologies, Santa Clara, CA, USA). The effect of HDAC inhibitor SAHA (Sigma Aldrich, St. Louis, MO, USA) on cell metabolism was also evaluated. The 80,000 cells were seeded in the Seahorse XFp culture plates and 1 µM SAHA was added 3 days before OCR or ECAR measurement. After the measurement, all samples were lysed with cell lysis buffer (50 mM Tris HCl pH = 6.8, 10% glycerol, 1% SDS), centrifuged at 20,000× *g* for 15 min at +4 °C, and protein concentration was determined with Pierce™ Modified Lowry Protein Assay Kit (Thermo Fisher Scientific, Waltham, MA, USA). Energetic parameters were normalized by protein concentration and expressed as (pM)/protein (mg/mL) for OCR and (mpH/protein (mg/mL) for ECAR measurements.

### 4.9. Western Blotting

Approximately 2 × 10^5^ cells were lysed with Radioimmunoprecipitation assay buffer (RIPA) buffer (150 mM NaCl, 5 mM EDTA, 50 mM Tris, 1% nonyl phenoxypolyethoxylethanol (NP-40), 0.5% sodium deoxycholate, 0.1% SDS, 1 tablet of protease inhibitors for 50 mL of RIPA buffer (A32963, Thermo Fisher Scientific, Waltham, MA, USA), 1 M NaF, 100 mM phenylmethylsulfonyl fluoride (PMSF), 200 mM sodium orthovanadate (Na_3_VO_4_)). Protein concentration was determined with Pierce™ Modified Lowry Protein Assay Kit (Thermo Fisher Scientific, Waltham, MA, USA). Protein levels were equated and an equal amount of protein for all samples was prepared for Western blotting. Protein samples were denatured with 6× sample buffer (375 mM Tris HCl pH 6.8, 6% SDS, 48% glycerol, 9% 2-mercaptoethanol, 0.03% bromophenol blue) and heated at 95 °C for 5 min. SDS-PAGE was performed using 4–12% gradient gels (Thermo Fisher Scientific, Waltham, MA, USA) and proteins were transferred to polyvinylidene fluoride (PVDF) membrane (Thermo Fisher Scientific, Waltham, MA, USA) under standard conditions. Membrane was washed with TBST (Tris buffered saline with 20% Tween 20) and blocked with 3% BSA in TBST for 1 h at room temperature. Primary alpha cardio actin antibody (GTX101876, GeneTex, Irvine, CA, USA) or beta actin antibody (sc-47778, Santa Cruz Biotechnology, Dallas, TX, USA) was added to the membrane and incubated overnight at 4 °C, washed with TBST 4 × 5 min, and probed with horseradish peroxidase (HRP)-conjugated secondary antibody (Thermo Fisher Scientific, Waltham, MA, USA) under at room temperature (RT) for 1 h. The membrane was washed 3 × 5 min with TBST. SuperSignal West Pico chemiluminescent substrate (Thermo Fisher Scientific, Waltham, MA, USA) was added to detect probed proteins. Signal detection and fixation was done using Kodak developer and fixer (Kodak, Rochester, NY, USA).

### 4.10. Immunocytochemistry

Healthy and pathological hmMSC were seeded on glass coverslips and grown until fully confluent. Cardiac differentiation was induced in Dulbecco’s modified Eagle’s medium (DMEM)/F12 (Thermo Fisher Scientific, Waltham, MA, USA) with 2% FBS medium (Thermo Fisher Scientific, Waltham, MA, USA) and 1 µM SAHA. Cells were differentiated for 3, 7, and 14 days, fixed with 4% paraformaldehyde at room temperature for 15 min, permeabilized with 0.1% Triton-100 for 15 min, and incubated with alpha cardio actin primary antibody (GTX101876, GeneTex, Irvine, CA, USA) and beta actin (sc8432, Santa Cruz Biotechnology, Dallas, TX, USA) overnight. Alexa Fluor 488-conjugated antibodies were used as secondary anti-rabbit antibodies (A16096, Thermo Fisher Scientific, Waltham, MA, USA) and anti-mouse (A16160, Thermo Fisher Scientific, Waltham, MA, USA). Additionally, cells’ nuclei were stained with 1 µg/mL of 4′,6-Diamidine-2′-phenylindole dihydrochloride (DAPI) for 2 min. Confocal images were acquired with confocal laser scanning microscope Leica TCS SP8 (Leica Microsystems, Wetzlar, Germany).

### 4.11. Electron Microscopy

Adherent cells were trypsinized using 0.25% Trypsin-EDTA (Thermo Fisher Scientific, Waltham, MA, USA). Cells were fixed twice, firstly with 2% glutaraldehyde (Sigma Aldrich, St. Louis, MO, USA) solution to stabilize proteins and secondly with osmium tetroxide (Sigma Aldrich, St. Louis, MO, USA) stabilize lipids. Latter cells were dehydrated with increasing ethanol (Sigma Aldrich, St. Louis, MO, USA) and propylene oxide (Sigma Aldrich, St. Louis, MO, USA) concentration. Fully dehydrated samples were infused with epoxy resin (Sigma Aldrich, St. Louis, MO, USA), cut on Leica UC6 ultramicrotome, and contrasted with 4% uranyl acetate and 3% lead citrate. Mitochondria and other organelle morphology was analyzed with transmission electron microscope JEM-100B (JEOL, Tokyo, Japan). 

### 4.12. Statistics

Statistical analysis was performed using Excel (Microsoft Corporation, Redmont, WA, USA) and Graphpad Prism 6.01 (GraphPad Software, San Diego, CA, USA) programs. Data are presented as means ± standard deviation (mean ± SD) from not less than 3–7 repeats of 2–3 independent experiments. In all experiments, cells from 2–3 healthy and dilated human myocardia were investigated. Results are presented as data ± SD from not less than 3–5 repeats. Data were significant at * *p* ≤ 0.05 or ** *p* ≤ 0.01 evaluated by calculation of student *t*-test parameter. 

### 4.13. Ethical Approval

The study was approved and permission was obtained from Vilnius Regional Biomedical Research Ethics Committee (License No. 158200-14-741-257, valid from l0.06.2014 until 30.06.2024). All patients gave written informed consent to investigate heart samples. The investigation conforms to the principles outlined in the Declaration of Helsinki.

## 5. Conclusions

In summary, data of this study show that SAHA inhibited expression of class I and II HDACs, downregulated glycolytic activity, and improved mitochondrial activity in both healthy and dilated myocardium-derived hmMSC, especially by improving mitochondrial membrane potential, decreasing proton leak, and improving coupling of respiration and ATP production. Better energetic status of the cells can strengthen the effect of HDAC inhibitors in terms of expression of cardio specific proteins essential for heart regeneration and proper functioning. Data of this study for the first time demonstrate the dual effect (suppression of HDAC activity and improved cell energetic homeostasis) of HDAC inhibitor SAHA on hmMSC resulting in better cardiomyogenic differentiation of dilated hmMSC that could improve functioning of heart tissue. Data of this study also show that dilated myocardium-derived cells still have cardiac regeneration potential, which can be purposefully stimulated. Application of HDAC inhibitors for the stimulation of regeneration potential of dilated myocardium-derived cells can be a promising therapeutic means for better heart functioning.

## Supplementary Materials

Supplementary materials can be found at https://www.mdpi.com/1422-0067/21/14/4845/s1.

## Abbreviations

DCM	Dilated cardiomyopathy
HDAC	Histone deacetylase
JC1	5,5′,6,6′-tetrachloro-1,1′,3,3′-tetraethylbenzimi- dazolylcarbocyanine iodide
HDACi	Histone deacetylase inhibitor
SAHA	Suberoylanilide hydroxamic acid
ATP	Adenosine triphosphate
iPS	Induced pluripotent stem cells
RNA	Ribonucleic acid
ECAR	Extracellular acidification
OCR	Oxygen consumption rate
FCCP	Carbonyl cyanide-4-(trifluoromethoxy)phenylhydrazone
2-DG	2-Deoxy-D-glucose
ACTC1	Actin alpha cardiac muscle 1
ACTB	Actin beta
GAPDH	Glyceraldehyde 3-phosphate dehydrogenase
DAPI	4′,6-diamidino-2-phenylindole
ROS	Reactive oxygen species
