# Supplementary material for: Histone Deacetylase Inhibitor Suberoylanilide Hydroxamic Acid Improves Energetic Status and Cardiomyogenic Differentiation of Human Dilated Myocardium-Derived Primary Mesenchymal Cells
Source: Int J Mol Sci. 2020 Jul 8;21(14):4845. doi: 10.3390/ijms21144845 (PMC7402340; doi:10.3390/ijms21144845)
Supplement: Supplementary file 1 [file ijms-21-04845-s001.pdf]

Supplementary Material

Histone Deacetylase Inhibitor Suberoylanilide Hydroxamic Acid Improves Energetic Status and Cardiomyogenic Differentiation of Human Dilated Myocardium-Derived Primary Mesenchymal Cells

Rokas Miksiunas [1], Kestutis Rucinskas [2], Vilius Janusauskas [2], Siegfried Labeit [3,4] and Daiva Bironaite [1,*]

Figure S1

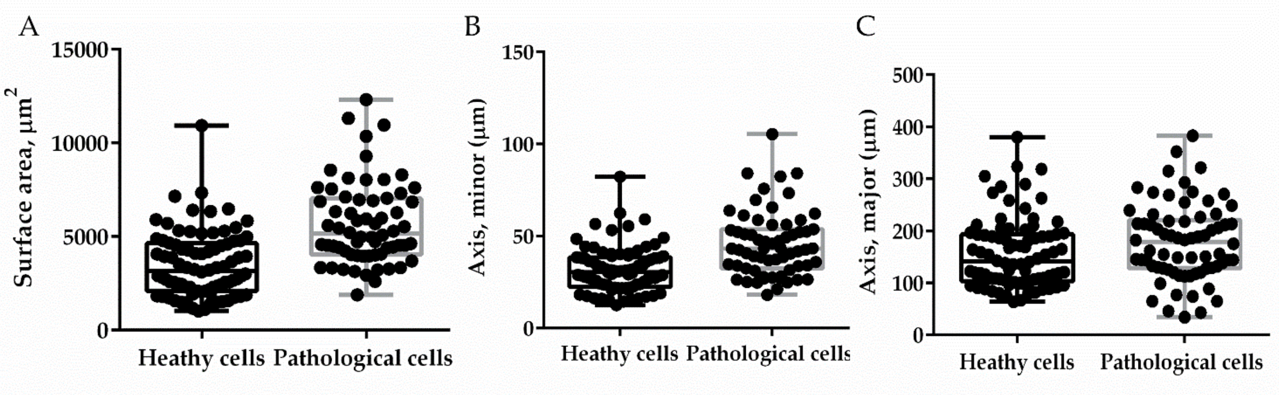

Figure S1. Graphical representation of total attachment area, minor and major cell axes of all healthy and pathological hmMSCs. A. Attachment area of healthy and pathological cell; **B.** Length of minor axes of the healthy and pathological hmMSC; **C.** Length of major axes of the healthy and pathological hmMSC. The distribution of 60 cells of each type are presented. Data are presented as mean ± SD.

Figure S2

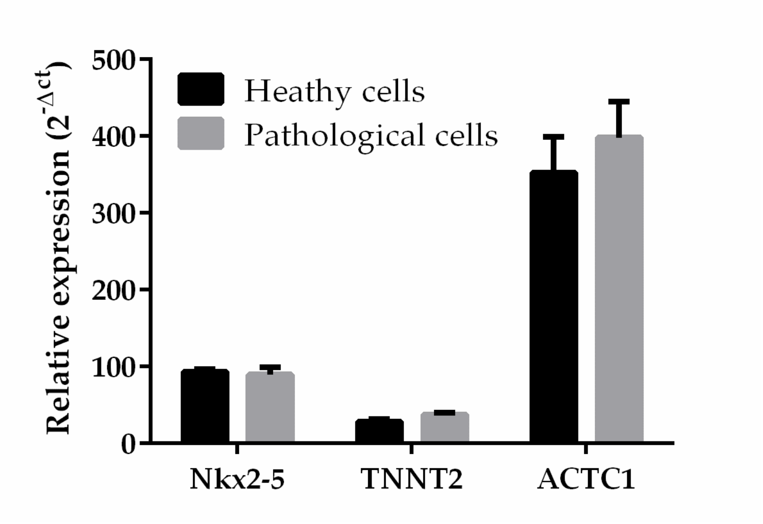

Figure S2. Expression of Nkx2-5, TNNT2 and ACTC1 at control levels. Gene expressions were normalized to average of housekeeping genes ACTB and GAPDH using 2^-ΔCT method. Data are presented as mean ± SD.
